# Utility of dynamic contrast enhancement for clinically significant prostate cancer detection

**DOI:** 10.1002/bco2.415

**Published:** 2024-08-04

**Authors:** Eric V. Li, Sai K. Kumar, Jonathan A. Aguiar, Mohammad R. Siddiqui, Clayton Neill, Zequn Sun, Edward M. Schaeffer, Anugayathri Jawahar, Ashley E. Ross, Hiten D. Patel

**Affiliations:** ^1^ Department of Urology, Feinberg School of Medicine Northwestern University Chicago Illinois USA; ^2^ Department of Preventive Medicine‐Division of Biostatistics Northwestern University Feinberg School of Medicine Chicago Illinois USA; ^3^ Department of Radiology, Feinberg School of Medicine Northwestern University Chicago Illinois USA

**Keywords:** diagnosis, dynamic contrast enhancement, nomogram, prostate cancer, prostate MRI, risk stratification

## Abstract

**Objective:**

This study aimed to evaluate the association of dynamic contrast enhancement (DCE) with clinically significant prostate cancer (csPCa, Gleason Grade Group ≥2) and compare biparametric magnetic resonance imaging (bpMRI) and multiparametric MRI (mpMRI) nomograms.

**Subjects/patients and methods:**

We identified a retrospective cohort of biopsy naïve patients who underwent pre‐biopsy MRI separated by individual MRI series from 2018 to 2022. csPCa detection rates were calculated for patients with peripheral zone (PZ) lesions scored 3–5 on diffusion weighted imaging (DWI) with available DCE (annotated as − or +). bpMRI Prostate Imaging Reporting and Data System (PIRADS) (3 = 3−, 3+; 4 = 4−, 4+; 5 = 5−, 5+) and mpMRI PIRADS (3 = 3−; 4 = 3+, 4−, 4+; 5 = 5−, 5+) approaches were compared in multivariable logistic regression models. Nomograms for detection of csPCa and ≥GG3 PCa incorporating all biopsy naïve patients who underwent prostate MRI were generated based on available serum biomarkers [PHI, % free prostate‐specific antigen (PSA), or total PSA] and validated with an independent cohort.

**Results:**

Patients (*n* = 1010) with highest PIRADS lesion in PZ were included in initial analysis with 127 (12.6%) classified as PIRADS 3+ (PIRADS 3 on bpMRI but PIRADS 4 on mpMRI). On multivariable analysis, PIRADS 3+ lesions were associated with higher csPCa rates compared to PIRADS 3− (3+ vs. 3−: OR 1.86, *p* = 0.024), but lower csPCa rates compared to PIRADS DWI 4 lesions (4 vs. 3+: OR 2.39, *p* < 0.001). csPCa rates were 19% (3−), 31% (3+), 41.5% (4−), 65.9% (4+), 62.5% (5−), and 92.3% (5+). bpMRI nomograms were non‐inferior to mpMRI nomograms in the development (*n* = 1410) and independent validation (*n* = 353) cohorts. Risk calculators available at: https://rossnm1.shinyapps.io/MynMRIskCalculator/.

**Conclusion:**

While DCE positivity by itself was associated with csPCa among patients with highest PIRADS lesions in the PZ, nomogram comparisons suggest that there is no significant difference in performance of bpMRI and mpMRI. bpMRI may be considered as an alternative to mpMRI for prostate cancer evaluation in many situations.

## 1 INTRODUCTION

Pre‐biopsy multiparametric prostate magnetic resonance imaging (mpMRI) increases detection of clinically significant prostate cancer (csPCa, defined as ISUP Grade Group ≥2), while avoiding unnecessary biopsies.^1^, ^2^ However, with an estimated 299 010 new cases of prostate cancer in the United States in 2024, there is increasing demand for diagnostic prostate MRI, which requires significant financial resources, availability of scanners, and qualified readers.^3^, ^4^ The average cost for mpMRI in the United States is $4400 with significant differences in charges among the facilities within the United States, and also varies widely internationally.^5^ Universally implementing mpMRI prior to biopsy decision significantly increases cost and could worsen healthcare disparities by creating unequal access to care.

Biparametric MRI (bpMRI) may enhance the accessibility to MRI assessments with reduced examination times, elimination of need for intravenous (IV) access, and avoidance of adverse events associated with gadolinium contrast.^6^ bpMRI primarily relies on T2 and diffusion weighted imaging (DWI) while omitting the dynamic contrast enhancement (DCE) phase, which is mainly used to upgrade peripheral zone (PZ) Prostate Imaging Reporting and Data System (PIRADS 3) lesions to PIRADS 4 lesions in PIRADS v2.1; DCE can also serve as a back‐up sequence if other sequences are inadequate (i.e. in setting of hip replacement) or for evaluation of recurrent prostate cancer.^7^ Early studies have suggested that bpMRI may be non‐inferior to mpMRI in terms of discriminative ability in csPCa detection, questioning the utility of DCE.^8^, ^9^ However, transitioning to bpMRI from mpMRI would proportionally increase PIRADS 3 lesions and decrease PIRADS 4 lesions in the PZ, changing overall csPCa risk stratification by PIRADS and utility of risk calculators in guiding the decision for biopsy.^10^, ^11^, ^12^ PIRADS DWI 3 patients upgraded by DCE to PIRADS 4 (called 3+) and PIRADS DWI 3 lesions with negative DCE (called 3−) are especially relevant for comparing bpMRI and mpMRI.

Therefore, we sought to (1) determine the association of DCE with csPCa detection by comparing risk‐stratification based on bpMRI, mpMRI (PIRADS v2.1), and a fully DCE stratified approach among patients with their highest PIRADS lesion in the PZ. Additionally, we evaluate (2) the impact of DCE on individualized risk calculation for all‐comers receiving MRI prior to biopsy by developing bpMRI‐based nomograms and comparing performance to previously developed mpMRI‐based nomograms.^10^

## 2 SUBJECTS/PATIENTS AND METHODS

### 2.1 Study populations

#### 2.1.1 Evaluation of DCE for csPCa detection in PZ PIRADS lesions

Between March 2018 and February 2022, we identified biopsy naïve patients who underwent pre‐biopsy MRI evaluated across the eleven hospital Northwestern Medicine healthcare system with IRB approval (STU00214996). The initial cohort of interest included patients receiving mpMRI before prostate biopsy with the highest PIRADS lesion in the PZ (PIRADS 3–5). Patients with transitional zone lesions as their highest PIRADS score, as well as patients with PIRADS 1 and 2 findings were excluded from the initial analysis. Patients with non‐contrast MRI study (*n* = 46), lack of DWI rating due to poor imaging quality (*n* = 23), or discrepancies in individual MRI series and final PIRADS rating (i.e. DWI score of 3 with positive DCE in PZ but PIRADS 3 recorded on final impression, *n* = 33) were excluded.

#### 2.1.2 bpMRI vs. mpMRI nomogram comparison

For bpMRI nomogram generation, we identified and included all biopsy naïve patients presenting for evaluation for elevated prostate‐specific antigen (PSA) 2–20 ng/mL receiving mpMRI as previously described for the generation of a mpMRI nomogram.^10^ The development cohort presented March 2018 to June 2021, and the independent validation cohort presented June 2021 to February 2022. As our objective was to assess performance in all‐comers evaluated by MRI for consideration of biopsy, patients with no discrete lesions (PIRADS 1 or 2), highest PIRADS in the transition zone, or deemed to be at low risk with PIRADS 1–3 and selected to forgo biopsy were included as previously described for development of the mpMRI‐based nomograms incorporating advanced serum biomarkers when available (PHI and % free PSA).^10^ There was a low rate of csPCa detection (2.4% at 23 months follow up) for patients selected to forgo biopsy with PIRADS 1–3 (Li et al, under review).

### 2.2 Screening strategy

Patients in our system are typically screened with PSA, prostate health index (PHI), and mpMRI.^10^ Biopsy decision is ultimately left to the provider and patient after consideration with other clinical factors. Baseline clinical characteristics, mpMRI reports, and prostate biopsy pathology reports were obtained. Continuous variables were categorized based on prior convention as previously described.

### 2.3 MRI evaluation

**FIGURE 1:**
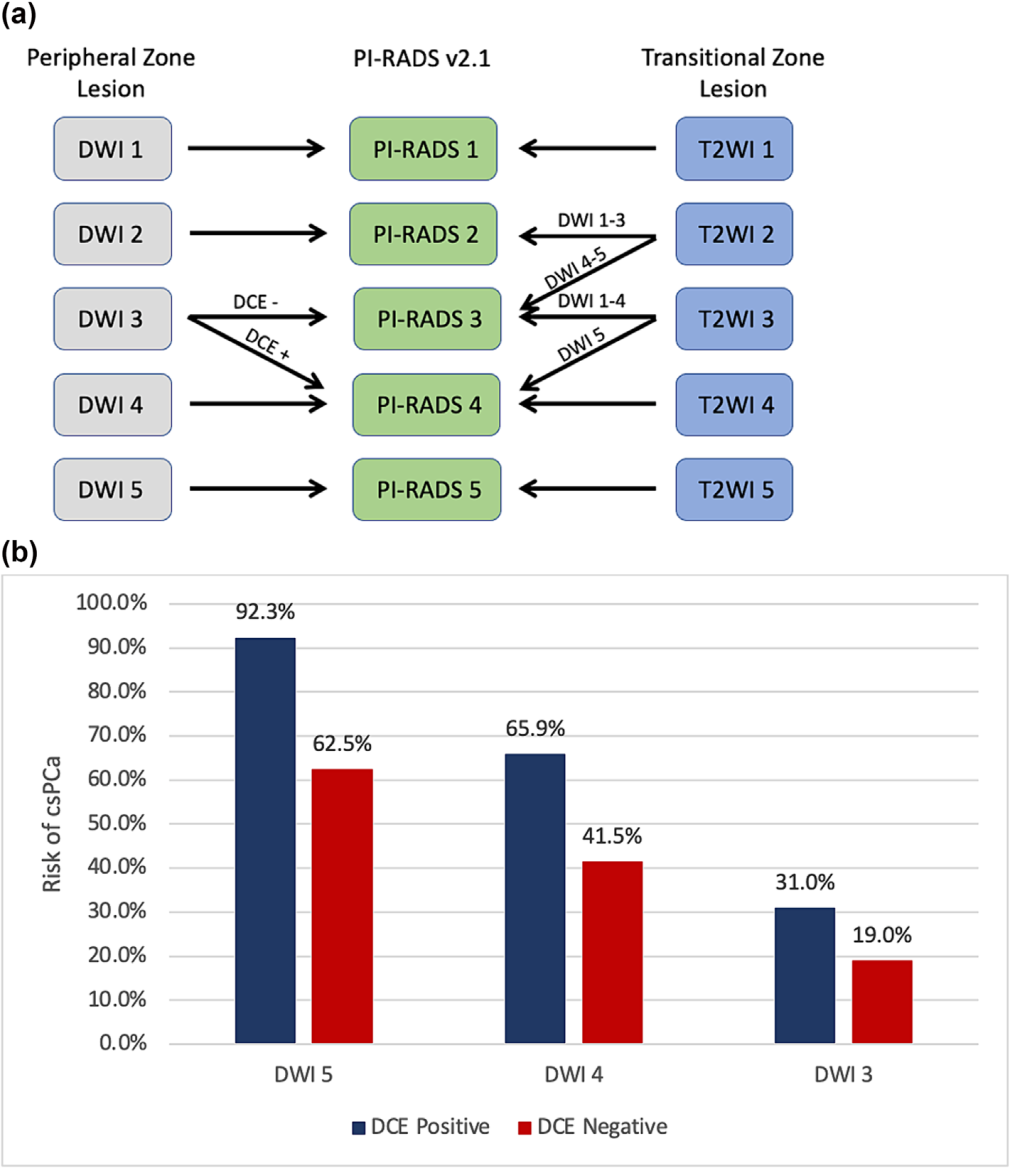
Categorization of (A) peripheral zone and transitional zone lesions with PI‐RADS v2.1 and (B) detection of csPCa by DWI score and full DCE stratification.

Patients undergoing mpMRI were given an assessment category using PIRADSv2.0 (before 2019) or v2.1 (2019 or later). mpMRI reports were reviewed and separated into T2, DWI, and DCE series with PIRADS score assigned for the T2 and DWI phases, while DCE was interpreted as positive (+) or negative (−). Patients with multiple lesions were categorized by highest PIRADS lesion. For this study, PIRADS DWI 3 lesions upgraded by DCE to PIRADS 4 were called 3+, PIRADS DWI 3 lesions with negative DCE were called 3−, and so on for PIRADS DWI 4 and PIRADS DWI 5 lesions. The bpMRI system categorizes patients into PIRADS 3 (3−, 3+), PIRADS DWI 4 (4−, 4+), and PIRADS DWI 5 (5−, 5+). The mpMRI system categorizes patients into PIRADS 3 (3−), PIRADS 4 (3+, 4−, 4+), and PIRADS 5 (5−, 5+) (Figure 1(A) for diagram). The fully DCE stratified approach considered PIRADS DWI 3−, 3+, 4−, 4+, 5−, and 5+ as six separate categories.

### 2.4 Prostate biopsy and pathological review

MRI‐guided biopsy was performed using cognitive or software fusion (UroNav platform, Phillips, Gainesville, FL). Suspicious lesions were biopsied with 2–5 cores, followed by systematic biopsies. Biopsy procedures were performed with transrectal or freehand transperineal approach with PrecisionPoint system (Perineologic, Cumberland, MD). MRI targeted biopsy fusion type and biopsy approach were determined per provider preference.

Pathology reports were reviewed for highest Gleason Grade and reported by highest International Society of Urological Pathology (ISUP) Gleason Grading system. Patients were categorized by overall highest Gleason Grade Group.

### 2.5 Study outcomes and statistical analysis

The primary outcome of interest for the initial analysis was detection of csPCa (≥GG2). We compared patients with PZ lesions classified as PIRADS DWI 3, 4, and 5 by DCE status (+ vs. −). Multivariable models evaluated performance of the original mpMRI PIRADS classification, a fully DCE stratified approach, and a bpMRI PIRADS DWI classification. For our bpMRI nomogram generation, we produced nomogram with outcomes of ≥GG2 and ≥GG3 PCa and developed various versions based on available serum biomarkers (PHI, % free PSA, or total PSA) using the same methods as previously described for mpMRI nomogram generation.^10^

Continuous variables were reported as median and interquartile range (IQR). Mann–Whitney *U*, Pearson's *T* test, and Chi squared tests were used to compare descriptive characteristics. Univariable and multivariable logistic regressions were performed to determine factors associated with csPCa detection. Total PSA and prostate volume were excluded from multivariable regression due to collinearity with prostate‐specific antigen density (PSAD). Indicator terms were utilized to compare DCE positive and negative lesions. To test discrimination, area under the curve (AUC) from receiver operating characteristic (ROC) curves were compared. Statistical significance was defined at *p* value <0.05, and statistical analyses were performed with R (version 4.2.1).

For nomogram generation, univariable and multivariable logistic regression models were used to identify factors associated with ≥GG2 and ≥GG3 prostate cancer for inclusion in nomogram using both biparametric and multiparametric parameters. ROC curves were generated, and DeLong's test was used to compare performance of bpMRI versus pMRI parameters. We then assessed our nomograms in the independent validation cohort, performing DCA and evaluating calibration of the model with Brier scores, Cox intercept, Cox slope, and Spiegelhalter *Z* statistics.

## 3 RESULTS

### 3.1 Evaluation of DCE for csPCa detection in PZ PIRADS lesions

#### 3.1.1 Baseline characteristics

**FIGURE 2:**
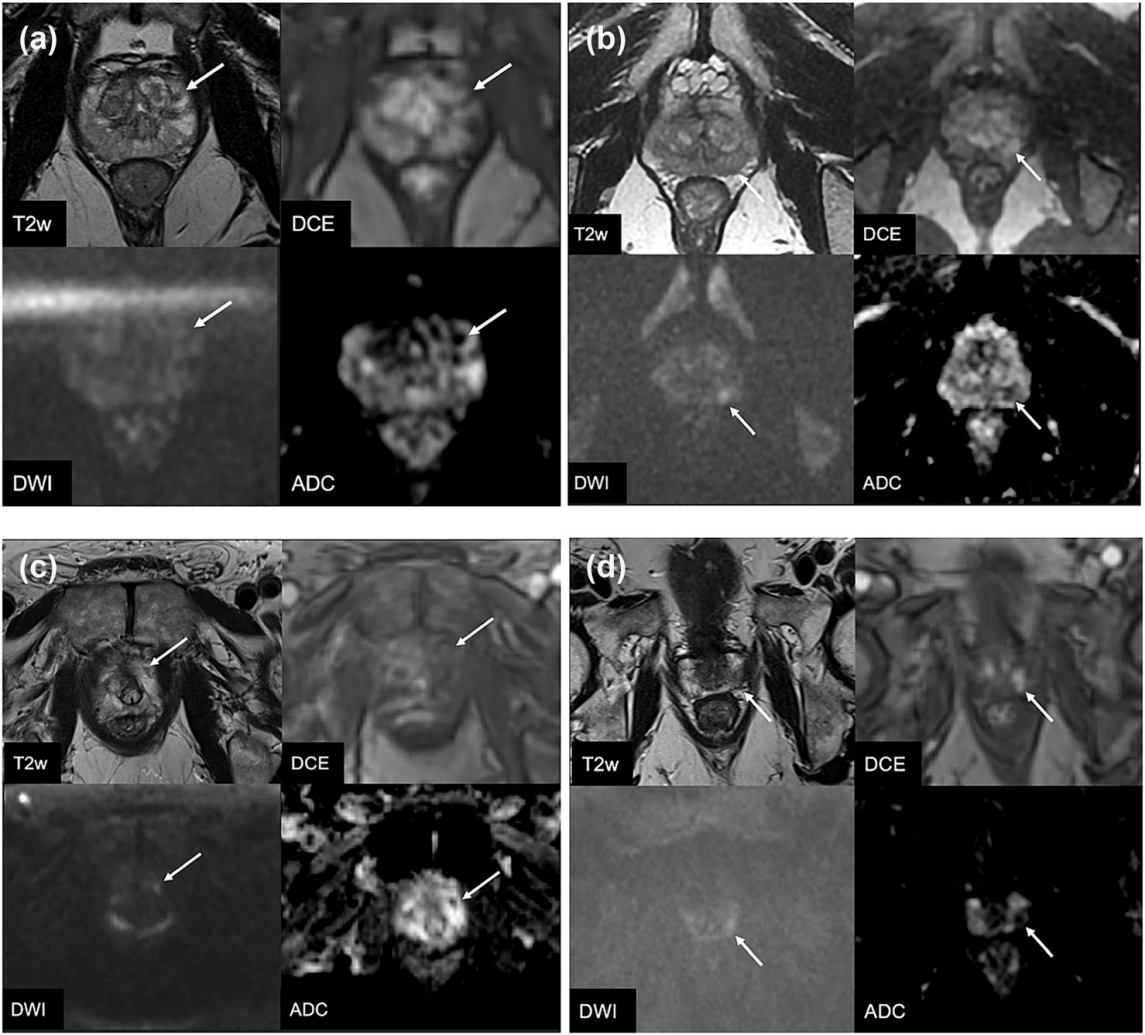
Representative MRI images of (A) PIRADS 3− with negative biopsy, (B) PIRADS 3+ with GG2 PCa on biopsy, (C) PIRADS 4− with negative biopsy, and (D) PIRADS 4+ with GG3 PCa on biopsy.

Patients (*n* = 1010) met inclusion criteria with highest PIRADS lesion in the PZ and underwent biopsy, with 53% (535/1010) diagnosed with csPCa. Figure 1(B) shows the stratification of csPCa detection by DWI and DCE. Figure 2 shows the MRI images of patients with PIRADS 3−, 3+, 4−, and 4+ lesions. DCE positivity was observed in 127/364 (34.9%), 305/452 (67.5%), and 196/212 (92.5%) among DWI 3, 4, and 5 patients. Overall, 127 (12.6%) patients were PIRADS 3+ and would be classified as PIRADS 4 by mpMRI PIRADS criteria but PIRADS 3 with omission of DCE by bpMRI. csPCa detection rates for patients classified by mpMRI PIRADS 3 (3−), 4 (3+, 4−, 4+), and 5 (5−, 5+) were 19%, 52%, and 90%, respectively (Table 1). With further stratification by DCE, patients with PIRADS 3−, 3+, and 4 lesions had csPCa detection rates of 19%, 31%, and 58%, respectively (*p* < 0.001). csPCa rates with bpMRI PIRADS 3 (3−, 3+), 4 (4−, 4+), and 5 (5−, 5+) were 23.7%, 58%, and 90%, respectively. There was significantly higher csPCa detection for DWI 4 and 5 lesions that were DCE positive (DWI 4: 65.9% for 4+ vs 41.5% for 4−, *p* < 0.001; DWI 5: 92.3% for 5+ vs 62.5% for 5−, *p* = 0.002; Figure 1(B)).

**TABLE 1:** Baseline characteristics stratified by csPCa.

	No Clinically Significant Prostate Cancer (*n* = 475)^1^	Clinically Significant Prostate Cancer (*n* = 535)^1^	*P* value
Age (years)	61 (56, 67)	65 (57, 71)	**<0.001**
Black race (%)	48/475 (10%)	86/535 (16%)	**0.01**
Insurance status (Private vs. Medicare/Medicaid)	64% Private, 36% Medicare/Medicaid	50% Private, 50% Medicare/Medicaid	**<0.001**
BMI (kg/m^2^)	27.2 (24.8, 30.4)	27.6 (25.1, 30.9)	0.2
PSA (ng/mL)	5.06 (3.78, 6.76)	6.01 (4.4, 9.17)	**<0.001**
PHI	35.6 (28.7, 45.7)	52.8 (40.5, 72.8)	**<0.001**
PHI category			**<0.001**
0–26.9	78 (16%)	18 (3.3%)	
27–35.9	113 (24%)	56 (10%)	
36–54.9	144 (30%)	152 (28%)	
≥55	38 (8.0%)	197 (36%)	
Not available	102 (21%)	116 (22%)	
PSA density (ng/mL/cm^3^)	0.10 (0.07, 0.14)	0.16 (0.10, 0.25)	**<0.001**
PSA density category (ng/mL/cm^3^)			**<0.001**
≤ 0.10	263 (55%)	135 (25%)	
0.10–0.15	118 (25%)	111 (21%)	
0.15–0.2	48 (10%)	106 (20%)	
≥ 0.20	46 (9.7%)	183 (34%)	
Prostate volume (cm^3^)	54 (39, 71)	39 (30, 53)	**<0.001**
Max mpMRI PIRADS (% biopsy positive for csPCa)			**<0.001**
3	177 (81%)	42 (19%)	
4	277 (48%)	302 (52%)	
5	21 (10%)	191 (90%)	
Max PIRADS by DWI and DCE (% biopsy positive for csPCa)			**<0.001**
3− DWI 3, DCE negative	177 (81%)	42 (19%)	
3+ DWI 3, DCE positive	87 (69%)	40 (31%)	
4 DWI 4, any DCE	190 (42%)	262 (58%)	
5	21 (10%)	191 (90%)	

Abbreviations: BMI, body mass index; csPCa, clinically significant prostate cancer; DCE, dynamic contrast enhancement; DWI, diffusion weighted imaging; IQR, interquartile range; mpMRI, multiparametric magnetic resonance imaging; PHI, prostate health index; PIRADS, Prostate Imaging Reporting and Data System; PSA, prostate‐specific antigen.

^1^ Median (IQR); *n* (%).

#### 3.1.2 Multivariable logistic regression models

On multivariable analysis, age, Black race, PHI, PSAD, and DCE stratified PIRADS scores were associated with csPCa (Table 2). With multivariable adjustment, PIRADS 3+ lesions were associated with significantly higher csPCa rates compared to PIRADS 3− (OR 1.86, 95% CI 1.08–3.21 p = 0.024, respectively), but lower csPCa rates compared to PIRADS DWI 4 lesions (OR 2.39, 95% CI 1.55–3.85, *p* < 0.001, respectively). DCE was also independently associated with csPCa for DWI 4 and 5 lesions (4+ vs. 4−: OR 2.29, 95% CI 1.48–3.57, *p* < 0.001; 5+ vs. 5−: OR 6.78, 95% CI 1.91–22.8, *p* = 0.002). **Supplemental Table** **S1** shows multivariable logistic regression models with classification by bpMRI as well as a fully DCE stratified model. There was no significant difference in AUC for multivariable models comparing mpMRI PIRADS and bpMRI PIRADS (0.816 vs. 0.820, *p* = 0.4), and marginally improved discrimination for fully DCE stratified PIRADS compared to standard mpMRI PIRADS (0.826 vs. 0.816, *p* = 0.04; **Supplemental Table** **S2**).

**TABLE 2:** Univariable and multivariable analysis of factors associated with csPCa with mpMRI PIRADS stratified by dynamic contrast enhancement across PIRADS DWI 3 patients (3− and 3+).

	Univariable analysis		Multivariable analysis	
	OR (95% CI)	*P* value	OR (95% CI)	*P* value
Age (years)	1.05 (1.03–1.06)	**<0.001**	1.03 (1.00–1.06)	**0.03**
Black race	1.70 (1.17–2.50)	**0.006**	1.76 (1.10–2.82)	**0.02**
Insurance category		**<0.001**		>0.9
Medicare	Ref			
Medicaid	0.70 (0.38–1.3)	0.3	1.11 (0.50–2.45)	0.8
Private	0.55 (0.42–0.71)	**<0.001**	0.99 (0.64–1.54)	>0.9
BMI (kg/m^2^)	1.02 (0.99–1.05)	0.2		
PHI Category		**<0.001**		**<0.001**
0–26.9	Ref			
27–35.9	2.15 (1.19–4.01)	**0.013**	1.77 (0.93–3.49)	0.09
36–54.9	4.51 (2.63–8.12)	**<0.001**	2.89 (1.60–5.46)	**<0.001**
≥55	22.2 (12.2–42.4)	**<0.001**	6.65 (3.38–13.6)	**<0.001**
Not available	4.93 (2.82–9.00)	**<0.001**	2.33 (1.25–4.53)	**0.01**
PSAD Category		**<0.001**		**<0.001**
≤ 0.10	Ref	**<0.001**		
0.10–≤ 0.15	1.83 (1.32–2.56)	**<0.001**	1.43 (0.96–2.11)	0.08
0.15–≤ 0.2	4.30 (2.90–6.46)	**<0.001**	2.71 (1.71–4.32)	**<0.001**
≥ 0.20	7.75 (5.32–11.5)	**<0.001**	3.29 (2.06–5.28)	**<0.001**
Max PIRADS		**<0.001**		
3	Ref			
4	4.59 (3.19–6.75)	**<0.001**		
5	38.3 (22.3–68.8)	**<0.001**		
Max PIRADS^1^		**<0.001**		**<0.001**
3−	Ref		Ref	
3+^2^	1.94 (1.17–3.21)	**0.01**	1.86 (1.08–3.21)	**0.02**
4^2^	5.81 (3.99–8.62)	**<0.001**	4.52 (3.01–6.91)	**<0.001**
5	38.3 (22.3–68.8)	**<0.001**	19.7 (11.0–36.6)	**<0.001**

Abbreviations: BMI, body mass index; CI, confidence interval; csPCa, clinically significant prostate cancer; DCE, dynamic contrast enhancement; DWI, diffusion weighted imaging; IQR, interquartile range; mpMRI, multiparametric magnetic resonance imaging; OR, odds ratio; PHI, prostate health index; PIRADS, Prostate Imaging Reporting and Data System; PSAD, prostate‐specific antigen density.

^1^ PIRADS 3− (DWI 3 with negative DCE), PIRADS 3+ (DWI 3 with positive DCE). PIRADS 4 (all DWI 4 lesions regardless of DCE). PIRADS 5 (all DWI 5 lesions regardless of DCE).

^2^ Indicator term OR 2.39 (95% CI 1.55–3.85, *P* < 0.001) comparing 3+ with 4 on multivariable analysis.

### 3.2 bpMRI nomogram generation and comparison of mpMRI and bpMRI performance

#### 3.2.1 Discrimination

To determine the impact of DCE on all patients receiving MRI evaluation prior to biopsy, a series of bpMRI nomograms were developed. Baseline characteristics for 1410 patients in the development cohort are summarized in **Supplemental Tables** **S3–S4** stratifying by ≥GG2 and ≥GG3 detection. Multivariable models identified age, Black race, PSAD, advanced serum biomarkers (if available, PHI or % free PSA), and PIRADS using either mpMRI or bpMRI parameters as variables included in nomograms (**Supplemental Figures** **S1–S2** **, Supplemental Table** **S5–S16**). Model ROCs are displayed in **Supplemental Figures** **S3–S4** and show AUCs of 0.891–0.909 for mpMRI models and AUCs of 0.894–0.913 for bpMRI models in the development cohort. Comparing the full multivariable models, bpMRI models were non‐inferior to performance of mpMRI models, and the bpMRI models marginally outperformed the mpMRI models for % free PSA and ≥GG3 (bpMRI AUC 0.912 vs 0.906, *p* = 0.03) and total PSA and ≥GG3 (bpMRI AUC 0.909 vs 0.903, *p* = 0.04; **Supplemental Table** **S17**).

#### 3.2.2 Independent cohort validation, decision curve analysis, and calibration

Decision curve analysis was completed which showed similar net benefit for mpMRI and bpMRI models compared to biopsy for no patients and all patients across clinically actionable threshold probabilities (**Supplemental Figures** **S5–S6**).

Baseline characteristics of the independent validation cohort are summarized in **Supplemental Tables** **S18–S19**. AUC values ranged 0.900–0.929 for mpMRI models and 0.904–0.931 for bpMRI models with no significant difference in AUCs comparing mpMRI and bpMRI (Table 3).

**TABLE 3:** DeLong comparison of ROC models for mpMRI and bpMRI models for validation Cohort.

Model	mpMRI AUC (95% CI)	bpMRI AUC (95% CI)	*P* value
PHI and ≥GG2	0.929 (0.903–0.954)	0.931 (0.905–0.956)	0.7
PHI and ≥GG3	0.909 (0.878–0.94)	0.909 (0.877–0.941)	>0.9
% free PSA and ≥GG2	0.923 (0.896–0.95)	0.928 (0.901–0.954)	0.3
% free PSA and ≥GG3	0.905 (0.872–0.938)	0.908 (0.877–0.94)	0.5
Total PSA and ≥GG2	0.913 (0.884–0.942)	0.919 (0.89–0.947)	0.3
Total PSA and ≥GG3	0.900 (0.866–0.935)	0.904 (0.871–0.936)	0.5

Abbreviations: AUC, area under the curve; bpMRI, biparametric magnetic resonance imaging; mpMRI, multiparametric magnetic resonance imaging; PHI, prostate health index; PSA, prostate‐specific antigen; ROC, receiver operating characteristic.

Calibration of our models in the validation cohort showed modest overprediction of probability of ≥GG2 and ≥GG3 prostate cancer (**Supplemental Figure** **S7–S8**). Brier scores were low, suggesting non‐significant differences between predicted and observed probabilities. The Spiegelhalter*Z*‐test *p* values were not statistically significant, so no recalibration was performed (**Supplemental Table** **S20**). The final bpMRI models have been added to the previously published mpMRI nomogram (MynMRIskCalculator) at https://rossnm1.shinyapps.io/MynMRIskCalculator/.

## 4 DISCUSSION

bpMRI offers a faster, more accessible pre‐biopsy evaluation in the setting of increased adoption of prostate MRI.^3^ Among those with PZ lesions in our large biopsy naïve mpMRI cohort, DCE positivity was associated with csPCa. However, when considering all patients undergoing prostate MRI evaluation for ≥GG2 and ≥GG3 PCa, there was no significant difference in performance of bpMRI nomogram models compared to mpMRI models. Overall, our results suggest that with our current biopsy practices, bpMRI can be a reasonable alternative to mpMRI and may be further considered for clinical adoption within the US.

There is limited data on the granular csPCa detection rates stratifying by DCE. We found that patients classified as PIRADS 4 on mpMRI (PIRADS v2.1) were a heterogeneous group depending on DCE with csPCa detection of 31% for 3+, 41.5% for 4−, and 65.9% for 4+ lesions. PIRADS 4 lesions upgraded by DCE (3+) also had higher csPCa detection rates than patients with PIRADS 3− (19% vs. 31%). Greer and colleagues evaluated concordance of DCE positive and negative lesions with csPCa on whole mount radical prostatectomy (RP) specimens, finding that there were higher rates of csPCa when stratifying by DCE (67.8% DCE positive PIRADS 3 vs 40% DCE negative PIRADS 3, *p* = 0.02).^13^ In contrast, Choi and colleagues report no significant difference between PIRADS 3 DCE positive versus negative lesions on whole mount RP specimens.^14^ These two small series were limited to patients receiving RP for previously diagnosed prostate cancer, which may not translate to patients undergoing prostate biopsy for initial diagnosis. In our study, we found that DCE positivity was associated with increased csPCa detection rates among patients with PIRADS 3–5 PZ lesions.

However, when making decisions regarding clinical practice, one must consider the wider population that is being evaluated with a risk stratification tool. When nomogram models were generated in the wider biopsy naïve population, there was no significant difference in csPCa and ≥GG3 detection comparing bpMRI and mpMRI models, which is consistent with prior studies. One study that evaluated 497 patients from the PROMIS trial found that performance of bpMRI and mpMRI was similar, with no cases of ≥GG3 PCa missed with bpMRI while utilizing detailed transperineal prostate mapping.^15^ Similar findings were reported from the Goteborg prostate cancer screening 2 trial where cancer detection relying on positivity of bpMRI was non‐inferior to mpMRI (15.1% vs. 15.2%).^16^ Prior metanalyses have conflicting results on sensitivity and performance of bpMRI and mpMRI due to a high degree of heterogeneity in patient populations, outcomes, and study design.^17^, ^18^ However, there are a number of considerations with implementing bpMRI, such as biopsy decision for PIRADS 3 lesions, reader experience, image quality, patient selection, and pre‐test probability of csPCa.

Decision to biopsy PIRADS 3 lesions remains controversial as csPCa detection rates vary from 12% to 23.9%, and as a result practice patterns vary widely.^2^, ^19^, ^20^ In general, for providers following a biopsy‐all approach based on a positive MRI (PIRADS 3–5), detection rates for bpMRI and mpMRI will be the same based on the PIRADS v2.1 grading system. DCE is less relevant to initially identifying a lesion as it is to modifying the relative degree of suspicion for men with a known lesion. At our institution, 77% of PIRADS 3 lesions in biopsy naïve patients were biopsied in a diagnostic pathway with PHI prior to mpMRI, and using these practice patterns, and the risk calculators showed no difference in bpMRI and mpMRI performance.^10^ However, for providers who are less inclined to biopsy PIRADS 3 lesions, implementation of bpMRI may make forgoing biopsy more difficult and increase biopsy rates of PIRADS 3 lesions.

Our findings must be interpreted in the context of differential performance of bpMRI and mpMRI based on reader experience and image quality. There is a significant range in positive predictive value for PIRADS ≥3 lesions even with mpMRI images across centres, individual radiologists, and by biopsy performance by urologists.^21^, ^22^ The utility of DCE may differ by reader experience level with less relevance for more experienced readers but important to optimize sensitivity among less experienced readers.^23^, ^24^ Additionally, image quality becomes paramount with omission of DCE, and healthcare systems that attempt to incorporate bpMRI will need to implement recall or on‐table monitoring to ensure that T2 and DWI images are of sufficient quality. Incorporation of bpMRI may be more feasible at high volume MRI centres and in healthcare systems with a robust imaging quality control and patient recall protocols in place.

Furthermore, patient selection based on pre‐test probability for csPCa and imaging indication is important for implementation of bpMRI. The PIRADS steering committee released a commentary on bpMRI for biopsy naïve men, recommending initial risk stratification as low risk and very high risk patients may not benefit from contrast studies.^25^ Patients at low risk of csPCa will have high probability of negative MRI, and patients at high risk of csPCa with evidence of locally advanced cancers by DRE or with significantly elevated PSAs will almost certainly have MRI lesions and subsequently undergo biopsy. Contrast administration is most likely to have utility in patients with intermediate risk for csPCa, prior negative biopsies with unexplained persistent PSA elevations, and patients undergoing active surveillance with concerning PSA kinetics. In these cases, DCE may improve reader confidence, decrease number of indeterminate cases, and help guide MRI‐guided biopsy particularly for smaller lesions.

First, our study is limited by its retrospective cohort design. We attempted to minimize selection bias by including all biopsy naïve patients evaluated by prostate MRI, including those who were not biopsied and those with negative MRIs, to understand the broader implications of bpMRI. Second, readers were not blinded to DCE series, as the series was based on individual series ratings on radiology reports among patients who all underwent mpMRI. Our hospital system includes a mix of community hospitals and high volume readers, but as discussed, reader experience affects the discriminative ability of prostate MRI. Third, while our nomograms were validated with an independent institutional cohort, further external validation of our models is required to evaluate generalizability. This is especially relevant to test performance across a large cohort of MRI readers with variations in reading experience and volume.

Acknowledging these limitations, we report that there is no significant difference in csPCa and ≥GG3 PCa discrimination using bpMRI and mpMRI in our nomogram models. The benefit of mpMRI was only apparent with a fully DCE stratified scoring system in the subset of patients with highest PIRADS lesions in the PZ. bpMRI offers a reasonable alternative to mpMRI, and may be considered for wider adoption in the United States.

## AUTHOR CONTRIBUTIONS

Eric V. Li was responsible for conceptualization, methodology, formal analysis, data curation, writing—original draft, writing—review and editing, and visualization. Sai K. Kumar was responsible for conceptualization, methodology, formal analysis, writing—original draft, writing—review and editing, and visualization. Jonathan A. Aguiar was responsible for data curation, writing—original draft, and writing—review and editing. Mohammad R. Siddiqui was responsible for conceptualization, data curation, and writing—review and editing. Clayton Neill was responsible for resources, data curation, and writing—review and editing. Zequn Sun was responsible for methodology, formal analysis, writing—review and editing, and supervision. Edward M. Schaeffer was responsible for writing—review and editing and supervision. Anugayathri Jawahar was responsible for writing—review and editing, visualization, and supervision. Ashley E. Ross was responsible for conceptualization, methodology, writing—review and editing, and supervision. Hiten D. Patel was responsible for conceptualization, methodology, writing—review and editing, and supervision.

## CONFLICT OF INTEREST STATEMENT

EMS is a consultant for Atria Academy of Science and Medicine, Early Medical, Lantheus, Pfizer, and PinnacleCare Health Advisors. AER is a consultant for Astellas, Astra Zeneca, Bayer HealthCare Pharmaceuticals, BillionToOne, Janssen Biotech, Lantheus, Myovant, Novartis, Pfizer, and Veracyte. These relationships are not related to the contents of this manuscript.

## Supporting information


**Table S1:** Multivariable Analysis of Factors Predictive of csPCa with PIRADS Classification by Biparametric MRI (PIRADS DWI 3, 4, and 5) as well as a fully Dynamic Contrast Enhancement stratified Model (3‐, 3+, 4‐, 4+, 5‐, 5+).
**Table S2:** AUCs for original PIRADS, stratification by DCE, and bpMRI parameters.
**Table S3:** Baseline Characteristics of Development Cohort for csPCa.
**Table S4:** Baseline Characteristics of Development Cohort for ≥GG3 PCa.
**Table S5:** Final Multivariable Model for ≥GG2 PCa with PHI for mpMRI.
**Table S6:** Final Multivariable Model for ≥GG3 PCa with PHI for mpMRI.
**Table S7:** Final Multivariable Model for ≥GG2 PCa with PHI for bpMRI.
**Table S8:** Final Multivariable Model for ≥GG3 PCa with PHI for bpMRI.
**Table S9:** Final Multivariable Model for ≥GG2 PCa with % free PSA for mpMRI.
**Table S10:** Final Multivariable Model for ≥GG3 PCa with % free PSA for mpMRI.
**Table S11:** Final Multivariable Model for ≥GG2 PCa with % free PSA for bpMRI.
**Table S12:** Final Multivariable Model for ≥GG3 PCa with % free PSA for bpMRI.
**Table S13:** Final Multivariable Model for ≥GG2 PCa with total PSA for mpMRI.
**Table S14:** Final Multivariable Model for ≥GG3 PCa with total PSA for mpMRI.
**Table S15:** Final Multivariable Model for ≥GG2 PCa with total PSA for bpMRI.
**Table S16:** Final Multivariable Model for ≥GG3 PCa with total PSA for bpMRI.
**Table S17:** DeLong comparison of ROC models for mpMRI and bpMRI models for development cohort.
**Table S18:** Baseline Characteristics of Validation Cohort for ≥GG2 PCa.
**Table S19:** Baseline Characteristics of Validation Cohort for ≥GG3 PCa.
**Table S20:** Calibration Curve Test Characteristics.
**Figure S1:** Nomograms for Advanced serum biomarker for ≥GG2 PCa A. PHI and mpMRI B. % free PSA and mpMRI C. PSA and mpMRI D. PHI and bpMRI E. % free PSA and bpMRI F. PSA and bpMRI.
**Figure S2:** Nomograms for Advanced serum biomarker for ≥GG3 PCa A. PHI and mpMRI B. % free PSA and mpMRI C. PSA and mpMRI D. PHI and bpMRI E. % free PSA and bpMRI F. PSA and bpMRI.
**Figure S3:** Receiver Operating Characteristic Curves for ≥GG2 PCa.
**Figure S4:** Receiver Operating Characteristic Curves for ≥GG3 PCa.
**Figure S5:** Decision Curve Analysis for ≥GG2 PCa.
**Figure S6:** Decision Curve Analysis for ≥GG3 PCa.
**Figure S7:** Calibration Curves for ≥GG2 PCa.
**Figure S8:** Calibration Curves for ≥GG3 PCa.
