# Supplementary material for: Utility of dynamic contrast enhancement for clinically significant prostate cancer detection
Source: BJUI Compass. 2024 Aug 4;5(9):865–73. doi: 10.1002/bco2.415 (PMC11420102; doi:10.1002/bco2.415)

**Supplemental Table 1: Multivariable Analysis of Factors Predictive of csPCa with PIRADS Classification by Biparametric MRI (PIRADS DWI 3, 4, and 5) as well as a fully Dynamic Contrast Enhancement stratified Model (3-, 3+, 4-, 4+, 5-, 5+).**

|  | Biparametric MRI  Multivariable Analysis |  | Full DCE Stratification  Multivariable Analysis |  |
| --- | --- | --- | --- | --- |
|  | OR (95% CI) | P value | OR (95% CI) | P value |
| Age (years) | 1.03 (1.01-1.06) | **0.018** | 1.03 (1.00-1.05) | 0.067 |
| Black Race | 1.75 (1.10-2.81) | **0.018** | 1.66 (1.04-2.68) | **0.036** |
| Insurance category |  | >0.9 |  | >0.9 |
| Medicare | Ref |  | Ref |  |
| Medicaid | 1.10 (0.50-2.42) | 0.8 | 1.10 (0.49-2.45) | 0.8 |
| Private | 1.00 (0.65-1.55) | >0.9 | 0.96 (0.61-1.50) | 0.9 |
| PHI Category |  | **<0.001** |  | **<0.001** |
| 0-26.9 | Ref |  |  |  |
| 27-35.9 | 1.75 (0.92-3.44) | 0.094 | 1.66 (0.87-3.28) | 0.13 |
| 36-54.9 | 2.86 (1.58-5.39) | **<0.001** | 2.73 (1.50-5.16) | **0.001** |
| ≥55 | 6.29 (3.21-12.8) | **<0.001** | 6.12 (3.09-12.6) | **<0.001** |
| Not available | 2.30 (1.23-4.45) | **0.011** | 2.22 (1.18-4.33) | **0.015** |
| PSAD Category |  |  |  |  |
| ≤ 0.10 | Ref |  | Ref |  |
| 0.10-≤0.15 | 1.43 (0.97-2.11) | 0.07 | 1.45 (0.97-2.16) | 0.066 |
| 0.15-≤0.2 | 2.83 (1.79-4.51) | **<0.001** | 2.90 (1.82-4.66) | **<0.001** |
| ≥ 0.20 | 3.40 (2.14-5.46) | **<0.001** | 3.13 (1.95-5.07) | **<0.001** |
| Max PIRADS |  | **<0.001** | - | - |
| 3-/3+ | Ref |  | - | - |
| 4 | 3.50 (2.50-4.92) | **<0.001** | - | - |
| 5 | 15.2 (8.96-26.9) | **<0.001** | - | - |
| Max PIRADS DWI | - | - |  | **<0.001** |
| 3- | - | - | Ref |  |
| 3+ | - | - | 1.85 (1.08-3.19) | **0.025** |
| 4-^1^ | - | - | 2.64 (1.60-4.40) | **<0.001** |
| 4+^1^ | - | - | 6.04 (3.90-9.49) | **<0.001** |
| 5-^2^ | - | - | 3.88 (1.24-13.1) | **0.022** |
| 5+^2^ | - | - | 26.3 (14.0-52.5) | **<0.001** |

^1^ Indicator OR 2.29 (95% CI 1.48-3.57, p<0.001)

^2^ Indicator OR 6.78 (95% CI 1.91-22.8, p=0.002)

**Supplemental Table 2: AUCs for original PIRADS, stratification by DCE, and bpMRI parameters**

|  | Multivariable Model^1^  (95% CI) | P value^2^ |
| --- | --- | --- |
| Original mpMRI PIRADS Model | 0.816  (0.790-0.842) | - |
| mpMRI PIRADS Model with DCE Modification  (DWI 3-, 3+, 4) | 0.822  (0.797-0.847) | 0.10 |
| Fully DCE Stratified Model  (3-, 3+, 4-, 4+, 5-, 5+) | 0.826  (0.801-0.852) | **0.04** |
| bpMRI Model | 0.820  (0.795-0.846) | 0.4 |

**^1^Adjusted for age, Black race, PHI category, and PSA density**

**^2^Comparisons are compared with original PIRADS classification**

**Supplemental Table 3: Baseline Characteristics of Development Cohort for csPCa**

|  | No Clinically Significant Prostate Cancer  (n=952)^1^ | Clinically Significant Prostate Cancer  (n=458)^1^ | P value |
| --- | --- | --- | --- |
| Age (years) | 63  (56, 68) | 65  (58, 70) | **<0.001** |
| Black race (%) | 84/952 (8.8%) | 72/458 (16%) | **<0.001** |
| Insurance status (Private vs. Medicare/Medicaid) | 63% Private, 37% Medicare/Medicaid | 51% Private, 49% Medicare/Medicaid | **<0.001** |
| PSA category  (ng/mL) |  |  | **<0.001** |
| 2-4 | 300 (32%) | 81 (18%) |  |
| 4-10 | 581 (61%) | 306 (67%) |  |
| 10-20 | 71 (7.5%) | 71 (16%) |  |
| % Free PSA |  |  | **<0.001** |
| <10% | 72 (8.8%) | 111 (29%) |  |
| 10-15% | 152 (19%) | 114 (30%) |  |
| 15-20% | 218 (27%) | 82 (22%) |  |
| 20-25% | 190 (23%) | 45 (12%) |  |
| ≥25% | 185 (23%) | 27 (7.1%) |  |
| Not available | 135 | 79 |  |
| PHI category |  |  | **<0.001** |
| 0-26.9 | 186 (24%) | 14 (3.8%) |  |
| 27-35.9 | 262 (34%) | 57 (16%) |  |
| 36-54.9 | 266 (34%) | 141 (39%) |  |
| ≥55 | 64 (8.2%) | 152 (42%) |  |
| Not available | 174 | 94 |  |
| PSA density category  (ng/mL/ cm^3^) |  |  | **<0.001** |
| ≤ 0.10 | 635 (67%) | 120 (26%) |  |
| 0.10-0.15 | 199 (21%) | 110 (24%) |  |
| 0.15-0.2 | 61 (6.4%) | 90 (20%) |  |
| ≥ 0.20 | 57 (6%) | 138 (30%) |  |
| Max PIRADS with mpMRI parameters |  |  | **<0.001** |
| 1 or 2 | 461 (48%) | 20 (4.4%) |  |
| 3 | 268 (28%) | 54 (12%) |  |
| 4 | 198 (21%) | 236 (52%) |  |
| 5 | 25 (2.6%) | 148 (32%) |  |
| Max PIRADS with bpMRI parameters |  |  | **<0.001** |
| 1 or 2 | 461 (48%) | 20 (4.4%) |  |
| 3 | 326 (34%) | 80 (17%) |  |
| 4 | 140 (15%) | 210 (46%) |  |
| 5 | 25 (2.6%) | 148 (32%) |  |

^1^Median (IQR); n (%)

**Supplemental Table 4: Baseline Characteristics of Development Cohort for ≥GG3 PCa**

|  | No ≥GG3 PCa  (n=1151)^1^ | ≥GG3 PCa  (n=259)^1^ | P value |
| --- | --- | --- | --- |
| Age (years) | 63  (56, 68) | 66  (61, 72) | **<0.001** |
| Black race (%) | 112/1151 (9.7%) | 44/259 (17%) | **<0.001** |
| Insurance status (Private vs. Medicare/Medicaid) | 63% Private, 37% Medicare/Medicaid | 42% Private, 58% Medicare/Medicaid | **<0.001** |
| PSA category  (ng/mL) |  |  | **<0.001** |
| 2-4 | 346 (30%) | 35 (14%) |  |
| 4-10 | 714 (62%) | 173 (67%) |  |
| 10-20 | 91 (7.9%) | 51 (20%) |  |
| % Free PSA |  |  | **<0.001** |
| <10% | 115 (12%) | 68 (33%) |  |
| 10-15% | 200 (20%) | 66 (32%) |  |
| 15-20% | 262 (27%) | 38 (18%) |  |
| 20-25% | 214 (22%) | 21 (10%) |  |
| ≥25% | 197 (20%) | 15 (7.2%) |  |
| Not available | 163 | 51 |  |
| PHI category |  |  | **<0.001** |
| 0-26.9 | 195 (21%) | 5 (2.5%) |  |
| 27-35.9 | 298 (32%) | 21 (10%) |  |
| 36-54.9 | 334 (35%) | 73 (36%) |  |
| ≥55 | 114 (12%) | 102 (51%) |  |
| Not available | 174 | 94 |  |
| PSA density category  (ng/mL/ cm^3^) |  |  | **<0.001** |
| ≤ 0.10 | 695 (60%) | 60 (23%) |  |
| 0.10-0.15 | 260 (23%) | 49 (19%) |  |
| 0.15-0.2 | 99 (8.6%) | 52 (20%) |  |
| ≥ 0.20 | 97 (8.4%) | 98 (38%) |  |
| Max PIRADS with mpMRI parameters |  |  | **<0.001** |
| 1 or 2 | 476 (41%) | 5 (1.9%) |  |
| 3 | 309 (27%) | 13 (5%) |  |
| 4 | 307 (27%) | 128 (49%) |  |
| 5 | 59 (5.1%) | 114 (44%) |  |
| Max PIRADS with bpMRI parameters |  |  | **<0.001** |
| 1 or 2 | 476 (41%) | 5 (1.9%) |  |
| 3 | 385 (33%) | 21 (8.1%) |  |
| 4 | 231 (20%) | 119 (46%) |  |
| 5 | 59 (5.1%) | 114 (44%) |  |

^1^Median (IQR); n (%)

**Supplemental Table 5: Final Multivariable Model for ≥GG2 PCa with PHI for mpMRI**

| Characteristic | N | OR | 95% CI | P value |
| --- | --- | --- | --- | --- |
| Age (years) | 1,410 | 1.02 | 1.00, 1.04 | **0.04** |
| Race |  |  |  |  |
| Not Black | 1,254 | — | — |  |
| Black | 156 | 1.88 | 1.17, 3.02 | **0.009** |
| PSA density category  (ng/mL/ cm^3^) |  |  |  | **<0.001** |
| ≤ 0.10 | 755 | — | — |  |
| 0.10-0.15 | 309 | 1.93 | 1.32, 2.82 | **<0.001** |
| 0.15-0.2 | 151 | 3.88 | 2.43, 6.25 | **<0.001** |
| ≥ 0.20 | 195 | 4.60 | 2.84, 7.53 | **<0.001** |
| PHI category |  |  |  | **<0.001** |
| 0-26.9 | 200 | — | — |  |
| 27-35.9 | 319 | 2.28 | 1.17, 4.69 | **0.02** |
| 36-54.9 | 407 | 3.97 | 2.12, 7.91 | **<0.001** |
| ≥55 | 216 | 7.90 | 3.89, 16.9 | **<0.001** |
| Not available | 268 | 3.70 | 1.90, 7.61 | **<0.001** |
| Max PIRADS with mpMRI parameters |  |  |  | **<0.001** |
| 1 or 2 | 481 | — | — |  |
| 3 | 322 | 4.45 | 2.59, 7.92 | **<0.001** |
| 4 | 434 | 20.2 | 12.4, 34.5 | **<0.001** |
| 5 | 173 | 64.1 | 34.2, 126 | **<0.001** |

**Supplemental Table 6: Final Multivariable Model for ≥GG3 PCa with PHI for mpMRI**

| Characteristic | N | OR | 95% CI | P value |
| --- | --- | --- | --- | --- |
| Age (years) | 1,410 | 1.04 | 1.02, 1.07 | **<0.001** |
| Race |  |  |  |  |
| Not Black | 1,254 | — | — |  |
| Black | 156 | 1.80 | 1.08, 2.98 | **0.02** |
| PSA density category  (ng/mL/ cm^3^) |  |  |  | **<0.001** |
| ≤ 0.10 | 755 | — | — |  |
| 0.10-0.15 | 309 | 1.14 | 0.71, 1.84 | 0.6 |
| 0.15-0.2 | 151 | 2.54 | 1.52, 4.25 | **<0.001** |
| ≥ 0.20 | 195 | 3.69 | 2.24, 6.13 | **<0.001** |
| PHI category |  |  |  | **<0.001** |
| 0-26.9 | 200 | — | — |  |
| 27-35.9 | 319 | 1.77 | 0.66, 5.67 | 0.3 |
| 36-54.9 | 407 | 3.75 | 1.53, 11.4 | **0.008** |
| ≥55 | 216 | 5.52 | 2.17, 17.1 | **<0.001** |
| Not available | 268 | 4.03 | 1.59, 12.4 | **0.007** |
| Max PIRADS with mpMRI parameters |  |  |  | **<0.001** |
| 1 or 2 | 481 | — | — |  |
| 3 | 322 | 3.67 | 1.35, 11.6 | **0.02** |
| 4 | 434 | 25.9 | 11.4, 74.6 | **<0.001** |
| 5 | 173 | 78.6 | 32.9, 233 | **<0.001** |

**Supplemental Table 7: Final Multivariable Model for ≥GG2 PCa with PHI for bpMRI**

| Characteristic | N | OR | 95% CI | P value |
| --- | --- | --- | --- | --- |
| Age (years) | 1,410 | 1.02 | 1.00, 1.04 | 0.055 |
| Race |  |  |  |  |
| Not Black | 1,254 | — | — |  |
| Black | 156 | 2.01 | 1.25, 3.23 | **0.004** |
| PSA density category  (ng/mL/ cm^3^) |  |  |  | **<0.001** |
| ≤ 0.10 | 755 | — | — |  |
| 0.10-0.15 | 309 | 1.87 | 1.28, 2.74 | **0.001** |
| 0.15-0.2 | 151 | 4.20 | 2.61, 6.80 | **<0.001** |
| ≥ 0.20 | 195 | 4.87 | 3.00, 8.00 | **<0.001** |
| PHI category |  |  |  | **<0.001** |
| 0-26.9 | 200 | — | — |  |
| 27-35.9 | 319 | 2.32 | 1.18, 4.79 | **0.02** |
| 36-54.9 | 407 | 4.07 | 2.16, 8.13 | **<0.001** |
| ≥55 | 216 | 7.01 | 3.44, 15.0 | **<0.001** |
| Not available | 268 | 3.59 | 1.84, 7.41 | **<0.001** |
| Max PIRADS with bpMRI parameters |  |  |  | **<0.001** |
| 1 or 2 | 481 | — | — |  |
| 3 | 406 | 5.19 | 3.11, 9.03 | **<0.001** |
| 4 | 350 | 25.1 | 15.3, 43.4 | **<0.001** |
| 5 | 173 | 65.7 | 35.1, 129 | **<0.001** |

**Supplemental Table 8: Final Multivariable Model for ≥GG3 PCa with PHI for bpMRI**

| Characteristic | N | OR | 95% CI | P value |
| --- | --- | --- | --- | --- |
| Age (years) | 1,410 | 1.04 | 1.02, 1.07 | **<0.001** |
| Race |  |  |  |  |
| Not Black | 1,254 | — | — |  |
| Black | 156 | 1.92 | 1.14, 3.21 | **0.01** |
| PSA density category  (ng/mL/ cm^3^) |  |  |  | **<0.001** |
| ≤ 0.10 | 755 | — | — |  |
| 0.10-0.15 | 309 | 1.11 | 0.69, 1.80 | 0.7 |
| 0.15-0.2 | 151 | 2.63 | 1.56, 4.45 | **<0.001** |
| ≥ 0.20 | 195 | 3.87 | 2.33, 6.48 | **<0.001** |
| PHI category |  |  |  | **<0.001** |
| 0-26.9 | 200 | — | — |  |
| 27-35.9 | 319 | 1.79 | 0.66, 5.75 | 0.3 |
| 36-54.9 | 407 | 3.74 | 1.51, 11.4 | **0.009** |
| ≥55 | 216 | 4.80 | 1.88, 14.9 | **0.003** |
| Not available | 268 | 3.85 | 1.51, 11.9 | **0.009** |
| Max PIRADS with bpMRI parameters |  |  |  | **<0.001** |
| 1 or 2 | 481 | — | — |  |
| 3 | 406 | 4.49 | 1.78, 13.7 | **0.003** |
| 4 | 350 | 31.8 | 14.0, 91.9 | **<0.001** |
| 5 | 173 | 81.1 | 34.0, 241 | **<0.001** |

**Supplemental Table 9: Final Multivariable Model for ≥GG2 PCa with % free PSA for mpMRI**

| Characteristic | N | OR | 95% CI | P value |
| --- | --- | --- | --- | --- |
| Age (years) | 1,410 | 1.03 | 1.01, 1.06 | **0.001** |
| Race |  |  |  |  |
| Not Black | 1,254 | — | — |  |
| Black | 156 | 2.16 | 1.34, 3.50 | **0.002** |
| PSA density category  (ng/mL/ cm^3^) |  |  |  | **<0.001** |
| ≤ 0.10 | 755 | — | — |  |
| 0.10-0.15 | 309 | 1.87 | 1.28, 2.72 | **0.001** |
| 0.15-0.2 | 151 | 3.67 | 2.27, 5.98 | **<0.001** |
| ≥ 0.20 | 195 | 4.91 | 3.03, 8.05 | **<0.001** |
| % Free PSA Category |  |  |  | **<0.001** |
| <10% | 183 | — | — |  |
| 10-15% | 266 | 0.70 | 0.42, 1.18 | 0.2 |
| 15-20% | 300 | 0.58 | 0.34, 1.00 | 0.051 |
| 20-25% | 235 | 0.43 | 0.24, 0.79 | **0.007** |
| ≥25% | 212 | 0.22 | 0.11, 0.43 | **<0.001** |
| Not available | 214 | 0.67 | 0.38, 1.19 | 0.2 |
| Max PIRADS with mpMRI parameters |  |  |  | **<0.001** |
| 1 or 2 | 481 | — | — |  |
| 3 | 322 | 4.59 | 2.68, 8.16 | **<0.001** |
| 4 | 434 | 21.0 | 13.0, 35.8 | **<0.001** |
| 5 | 173 | 79.4 | 42.6, 155 | **<0.001** |

**Supplemental Table 10: Final Multivariable Model for ≥GG3 PCa with % free PSA for mpMRI**

| Characteristic | N | OR | 95% CI | P value |
| --- | --- | --- | --- | --- |
| Age (years) | 1,410 | 1.06 | 1.03, 1.08 | **<0.001** |
| Race |  |  |  |  |
| Not Black | 1,254 | — | — |  |
| Black | 156 | 1.89 | 1.13, 3.14 | **0.02** |
| PSA density category  (ng/mL/ cm^3^) |  |  |  | **<0.001** |
| ≤ 0.10 | 755 | — | — |  |
| 0.10-0.15 | 309 | 1.17 | 0.72, 1.89 | 0.5 |
| 0.15-0.2 | 151 | 2.45 | 1.45, 4.15 | **<0.001** |
| ≥ 0.20 | 195 | 3.94 | 2.38, 6.55 | **<0.001** |
| % Free PSA Category |  |  |  | **0.02** |
| <10% | 183 | — | — |  |
| 10-15% | 266 | 0.80 | 0.47, 1.36 | 0.4 |
| 15-20% | 300 | 0.57 | 0.31, 1.04 | 0.07 |
| 20-25% | 235 | 0.45 | 0.22, 0.91 | **0.03** |
| ≥25% | 212 | 0.33 | 0.15, 0.70 | **0.005** |
| Not available | 214 | 0.94 | 0.52, 1.69 | 0.8 |
| Max PIRADS with mpMRI parameters |  |  |  | **<0.001** |
| 1 or 2 | 481 | — | — |  |
| 3 | 322 | 3.83 | 1.41, 12.1 | **0.01** |
| 4 | 434 | 27.7 | 12.2, 79.5 | **<0.001** |
| 5 | 173 | 96.1 | 40.5, 285 | **<0.001** |

**Supplemental Table 11: Final Multivariable Model for ≥GG2 PCa with % free PSA for bpMRI**

| Characteristic | N | OR | 95% CI | P value |
| --- | --- | --- | --- | --- |
| Age (years) | 1,410 | 1.03 | 1.01, 1.05 | **0.003** |
| Race |  |  |  |  |
| Not Black | 1,254 | — | — |  |
| Black | 156 | 2.30 | 1.42, 3.73 | **<0.001** |
| PSA density category  (ng/mL/ cm^3^) |  |  |  | **<0.001** |
| ≤ 0.10 | 755 | — | — |  |
| 0.10-0.15 | 309 | 1.78 | 1.22, 2.61 | **0.003** |
| 0.15-0.2 | 151 | 3.88 | 2.38, 6.37 | **<0.001** |
| ≥ 0.20 | 195 | 4.96 | 3.04, 8.18 | **<0.001** |
| % Free PSA Category |  |  |  | **<0.001** |
| <10% | 183 | — | — |  |
| 10-15% | 266 | 0.73 | 0.43, 1.24 | 0.2 |
| 15-20% | 300 | 0.57 | 0.33, 0.99 | **0.045** |
| 20-25% | 235 | 0.46 | 0.25, 0.85 | **0.01** |
| ≥25% | 212 | 0.22 | 0.11, 0.44 | **<0.001** |
| Not available | 214 | 0.69 | 0.39, 1.23 | 0.2 |
| Max PIRADS with bpMRI parameters |  |  |  | **<0.001** |
| 1 or 2 | 481 | — | — |  |
| 3 | 406 | 5.32 | 3.19, 9.24 | **<0.001** |
| 4 | 350 | 27.0 | 16.4, 46.5 | **<0.001** |
| 5 | 173 | 80.9 | 43.3, 159 | **<0.001** |

**Supplemental Table 12: Final Multivariable Model for ≥GG3 PCa with % free PSA for bpMRI**

| Characteristic | N | OR | 95% CI | P value |
| --- | --- | --- | --- | --- |
| Age (years) | 1,410 | 1.05 | 1.03, 1.08 | **<0.001** |
| Race |  |  |  |  |
| Not Black | 1,254 | — | — |  |
| Black | 156 | 2.00 | 1.18, 3.35 | **0.009** |
| PSA density category  (ng/mL/ cm^3^) |  |  |  | **<0.001** |
| ≤ 0.10 | 755 | — | — |  |
| 0.10-0.15 | 309 | 1.11 | 0.68, 1.79 | 0.7 |
| 0.15-0.2 | 151 | 2.51 | 1.46, 4.31 | **<0.001** |
| ≥ 0.20 | 195 | 3.96 | 2.37, 6.67 | **<0.001** |
| % Free PSA Category |  |  |  | **0.03** |
| <10% | 183 | — | — |  |
| 10-15% | 266 | 0.84 | 0.48, 1.45 | 0.5 |
| 15-20% | 300 | 0.59 | 0.32, 1.08 | 0.09 |
| 20-25% | 235 | 0.50 | 0.24, 1.02 | 0.06 |
| ≥25% | 212 | 0.35 | 0.16, 0.76 | **0.009** |
| Not available | 214 | 1.01 | 0.56, 1.85 | >0.9 |
| Max PIRADS with bpMRI parameters |  |  |  | **<0.001** |
| 1 or 2 | 481 | — | — |  |
| 3 | 406 | 4.65 | 1.85, 14.2 | **0.002** |
| 4 | 350 | 34.6 | 15.2, 99.8 | **<0.001** |
| 5 | 173 | 98.0 | 41.3, 290 | **<0.001** |

**Supplemental Table 13: Final Multivariable Model for ≥GG2 PCa with total PSA for mpMRI**

| Characteristic | N | OR | 95% CI | P value |
| --- | --- | --- | --- | --- |
| Age (years) | 1,410 | 1.02 | 1.01, 1.04 | **0.01** |
| Race |  |  |  |  |
| Not Black | 1,254 | — | — |  |
| Black | 156 | 2.04 | 1.28, 3.26 | **0.003** |
| PSA density category  (ng/mL/ cm^3^) |  |  |  | **<0.001** |
| ≤ 0.10 | 755 | — | — |  |
| 0.10-0.15 | 309 | 2.11 | 1.46, 3.04 | **<0.001** |
| 0.15-0.2 | 151 | 4.84 | 3.08, 7.69 | **<0.001** |
| ≥ 0.20 | 195 | 7.03 | 4.48, 11.2 | **<0.001** |
| Max PIRADS with mpMRI parameters |  |  |  | **<0.001** |
| 1 or 2 | 481 | — | — |  |
| 3 | 322 | 4.52 | 2.64, 8.00 | **<0.001** |
| 4 | 434 | 21.7 | 13.5, 36.9 | **<0.001** |
| 5 | 173 | 82.0 | 44.2, 160 | **<0.001** |

**Supplemental Table 14: Final Multivariable Model for ≥GG3 PCa with total PSA for mpMRI**

| Characteristic | N | OR | 95% CI | P value |
| --- | --- | --- | --- | --- |
| Age (years) | 1,410 | 1.05 | 1.03, 1.07 | **<0.001** |
| Race |  |  |  |  |
| Not Black | 1,254 | — | — |  |
| Black | 156 | 1.86 | 1.12, 3.07 | **0.015** |
| PSA density category  (ng/mL/ cm^3^) |  |  |  | **<0.001** |
| ≤ 0.10 | 755 | — | — |  |
| 0.10-0.15 | 309 | 1.27 | 0.80, 2.02 | 0.3 |
| 0.15-0.2 | 151 | 3.06 | 1.87, 5.03 | **<0.001** |
| ≥ 0.20 | 195 | 5.19 | 3.25, 8.35 | **<0.001** |
| Max PIRADS with mpMRI parameters |  |  |  | **<0.001** |
| 1 or 2 | 481 | — | — |  |
| 3 | 322 | 3.86 | 1.43, 12.2 | **0.01** |
| 4 | 434 | 29.5 | 13.0, 84.6 | **<0.001** |
| 5 | 173 | 102 | 42.9, 301 | **<0.001** |

**Supplemental Table 15: Final Multivariable Model for ≥GG2 PCa with total PSA for bpMRI**

| Characteristic | N | OR | 95% CI | P value |
| --- | --- | --- | --- | --- |
| Age (years) | 1,410 | 1.02 | 1.00, 1.04 | **0.02** |
| Race |  |  |  |  |
| Not Black | 1,254 | — | — |  |
| Black | 156 | 2.16 | 1.35, 3.46 | **0.001** |
| PSA density category  (ng/mL/ cm^3^) |  |  |  | **<0.001** |
| ≤ 0.10 | 755 | — | — |  |
| 0.10-0.15 | 309 | 2.01 | 1.39, 2.92 | **<0.001** |
| 0.15-0.2 | 151 | 5.10 | 3.22, 8.16 | **<0.001** |
| ≥ 0.20 | 195 | 7.09 | 4.50, 11.3 | **<0.001** |
| Max PIRADS with bpMRI parameters |  |  |  | **<0.001** |
| 1 or 2 | 481 | — | — |  |
| 3 | 406 | 5.24 | 3.16, 9.09 | **<0.001** |
| 4 | 350 | 27.7 | 16.9, 47.5 | **<0.001** |
| 5 | 173 | 83.6 | 45.0, 163 | **<0.001** |

**Supplemental Table 16: Final Multivariable Model for ≥GG3 PCa with total PSA for bpMRI**

| Characteristic | N | OR | 95% CI | P value |
| --- | --- | --- | --- | --- |
| Age (years) | 1,410 | 1.05 | 1.02, 1.07 | **<0.001** |
| Race |  |  |  |  |
| Not Black | 1,254 | — | — |  |
| Black | 156 | 1.98 | 1.19, 3.29 | **0.008** |
| PSA density category  (ng/mL/ cm^3^) |  |  |  | **<0.001** |
| ≤ 0.10 | 755 | — | — |  |
| 0.10-0.15 | 309 | 1.20 | 0.75, 1.91 | 0.4 |
| 0.15-0.2 | 151 | 3.08 | 1.86, 5.11 | **<0.001** |
| ≥ 0.20 | 195 | 5.17 | 3.22, 8.38 | **<0.001** |
| Max PIRADS with bpMRI parameters |  |  |  | **<0.001** |
| 1 or 2 | 481 | — | — |  |
| 3 | 406 | 4.72 | 1.88, 14.3 | **0.002** |
| 4 | 350 | 36.8 | 16.2, 106 | **<0.001** |
| 5 | 173 | 103 | 43.6, 306 | **<0.001** |

**Supplemental Table 17: DeLong comparison of ROC models for mpMRI and bpMRI models for development cohort**

| Model | mpMRI AUC (95% CI) | bpMRI AUC  (95% CI) | P value |
| --- | --- | --- | --- |
| PHI and ≥GG2 | 0.901  (0.884-0.917) | 0.903  (0.887-0.919) | 0.3 |
| PHI and ≥GG3 | 0.909  (0.892-0.927) | 0.913  (0.896-0.93) | 0.09 |
| % free PSA and ≥GG2 | 0.896  (0.879-0.913) | 0.899  (0.882-0.916) | 0.2 |
| % free PSA and ≥GG3 | 0.906  (0.888-0.924) | 0.912  (0.894-0.929) | **0.03** |
| Total PSA and ≥GG2 | 0.891  (0.874-0.908) | 0.894  (0.877-0.911) | 0.2 |
| Total PSA and ≥GG3 | 0.903  (0.886-0.921) | 0.909  (0.892-0.926) | **0.04** |

**Supplemental Table 18: Baseline Characteristics of Validation Cohort for ≥GG2 PCa**

^1^Median (IQR); n (%)

|  | No Clinically Significant Prostate Cancer  (n=236)^1^ | Clinically Significant Prostate Cancer  (n=117)^1^ | P value |
| --- | --- | --- | --- |
| Age (years) | 63  (57, 70) | 66  (60, 72) | **0.005** |
| Black race (%) | 19/236 (8.1%) | 18/117 (15%) | **0.03** |
| Insurance status (Private vs. Medicare/Medicaid) | 61% Private, 39% Medicare/Medicaid | 45% Private, 55% Medicare/Medicaid | **0.02** |
| % Free PSA |  |  | **<0.001** |
| <10% | 11 (4.7%) | 22 (19%) |  |
| 10-15% | 31 (13%) | 32 (27%) |  |
| 15-20% | 38 (16%) | 18 (15%) |  |
| 20-25% | 31 (13%) | 13 (11%) |  |
| ≥25% | 68 (29%) | 7 (6.0%) |  |
| Not available | 57 (24%) | 25 (21%) |  |
| PHI category |  |  | **<0.001** |
| 0-26.9 | 33 (14%) | 0 (0%) |  |
| 27-35.9 | 59 (25%) | 5 (4.3%) |  |
| 36-54.9 | 66 (28%) | 38 (32%) |  |
| ≥55 | 13 (5.5%) | 48 (41%) |  |
| Not available | 65 (28%) | 26 (22%) |  |
| PSA density category  (ng/mL/ cm^3^) |  |  | **<0.001** |
| ≤ 0.10 | 149 (63%) | 21 (18%) |  |
| 0.10-0.15 | 46 (19%) | 24 (21%) |  |
| 0.15-0.2 | 24 (10%) | 25 (21%) |  |
| ≥ 0.20 | 17 (7.2%) | 47 (40%) |  |
| Max PIRADS with mpMRI parameters |  |  | **<0.001** |
| 1 or 2 | 91 (39%) | 1 (0.9%) |  |
| 3 | 75 (32%) | 7 (6.0%) |  |
| 4 | 58 (25%) | 52 (44%) |  |
| 5 | 12 (5.1%) | 57 (49%) |  |
| Max PIRADS with bpMRI parameters |  |  | **<0.001** |
| 1 or 2 | 91 (39%) | 1 (0.9%) |  |
| 3 | 92 (39%) | 10 (8.5%) |  |
| 4 | 41 (17%) | 49 (42%) |  |
| 5 | 12 (5.1%) | 57 (49%) |  |

|  | No Clinically Significant Prostate Cancer  (n=287)^1^ | Clinically Significant Prostate Cancer  (n=66)^1^ | P value |
| --- | --- | --- | --- |
| Age (years) | 64  (57, 70) | 66  (61, 73) | **0.01** |
| Black race (%) | 30/287 (10%) | 7/66 (11%) | >0.9 |
| Insurance status (Private vs. Medicare/Medicaid) | 58% Private, 42% Medicare/Medicaid | 47% Private, 53% Medicare/Medicaid | 0.3 |
| % Free PSA |  |  | **<0.001** |
| <10% | 19 (6.6%) | 14 (21%) |  |
| 10-15% | 47 (16%) | 16 (24%) |  |
| 15-20% | 49 (17%) | 7 (11%) |  |
| 20-25% | 39 (14%) | 5 (7.6%) |  |
| ≥25% | 71 (25%) | 4 (6.1%) |  |
| Not available | 62 (22%) | 20 (30%) |  |
| PHI category |  |  | **<0.001** |
| 0-26.9 | 33 (11%) | 0 (0%) |  |
| 27-35.9 | 63 (22%) | 1 (1.5%) |  |
| 36-54.9 | 90 (31%) | 14 (21%) |  |
| ≥55 | 31 (11%) | 30 (45%) |  |
| Not available | 70 (24%) | 21 (32%) |  |
| PSA density category  (ng/mL/ cm^3^) |  |  | **<0.001** |
| ≤ 0.10 | 160 (56%) | 10 (15%) |  |
| 0.10-0.15 | 61 (21%) | 9 (14%) |  |
| 0.15-0.2 | 35 (12%) | 14 (21%) |  |
| ≥ 0.20 | 31 (11%) | 33 (50%) |  |
| Max PIRADS with mpMRI parameters |  |  | **<0.001** |
| 1 or 2 | 92 (32%) | 0 (0%) |  |
| 3 | 80 (28%) | 2 (3.0%) |  |
| 4 | 89 (31%) | 21 (32%) |  |
| 5 | 26 (9.1%) | 43 (65%) |  |
| Max PIRADS with bpMRI parameters |  |  | **<0.001** |
| 1 or 2 | 92 (32%) | 0 (0%) |  |
| 3 | 99 (34%) | 3 (4.5%) |  |
| 4 | 70 (24%) | 20 (30%) |  |
| 5 | 26 (9.1%) | 43 (65%) |  |

**Supplemental Table 19: Baseline Characteristics of Validation Cohort for ≥GG3 PCa**

^1^Median (IQR); n (%)

**Supplemental Table 20: Calibration Curve Test Characteristics**

|  | AUC | Brier Score | Cox (Calibration) Intercept | Cox (Calibration) Slope | Spiegelhalter Z statistic | Spiegelhalter Z statistic P value |
| --- | --- | --- | --- | --- | --- | --- |
| PHI and ≥GG2 mpMRI | 0.929 | 0.107 | -0.619 | 1.193 | -1.396 | 0.16 |
| PHI and ≥GG2 bpMRI | 0.931 | 0.104 | -0.666 | 1.19 | -1.365 | 0.17 |
| PHI and ≥GG3 mpMRI | 0.909 | 0.099 | -0.624 | 1.039 | -0.562 | 0.57 |
| PHI and ≥GG3 bpMRI | 0.909 | 0.100 | -0.648 | 1.035 | -0.371 | 0.71 |
| % free PSA and ≥GG2 mpMRI | 0.923 | 0.110 | -0.439 | 1.139 | -1.220 | 0.22 |
| % free PSA and ≥GG2 bpMRI | 0.928 | 0.106 | -0.484 | 1.150 | -1.312 | 0.19 |
| % free PSA and ≥GG3  mpMRI | 0.905 | 0.099 | -0.544 | 0.974 | -0.229 | 0.82 |
| % free PSA and ≥GG3  bpMRI | 0.908 | 0.098 | -0.570 | 0.980 | -0.154 | 0.88 |
| Total PSA and ≥GG2  mpMRI | 0.913 | 0.117 | -0.524 | 1.102 | -0.866 | 0.39 |
| Total PSA and ≥GG2  bpMRI | 0.919 | 0.113 | -0.561 | 1.111 | -0.991 | 0.32 |
| Total PSA and ≥GG3 mpMRI | 0.900 | 0.102 | -0.577 | 0.985 | -0.362 | 0.72 |
| Total PSA and ≥GG3 bpMRI | 0.904 | 0.101 | -0.601 | 0.985 | -0.176 | 0.86 |

**Supplemental Figure 1: Nomograms for Advanced serum biomarker for ≥GG2 PCa A. PHI and mpMRI B. % free PSA and mpMRI C. PSA and mpMRI D. PHI and bpMRI E. % free PSA and bpMRI F. PSA and bpMRI**

**
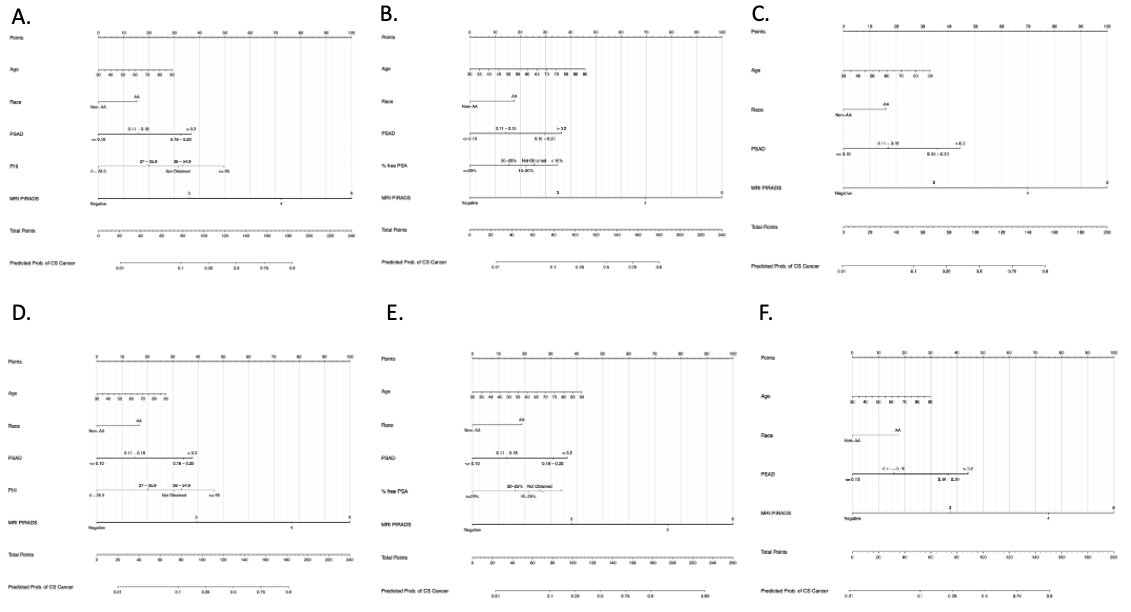
**

**Supplemental Figure 2: Nomograms for Advanced serum biomarker for ≥GG3 PCa A. PHI and mpMRI B. % free PSA and mpMRI C. PSA and mpMRI D. PHI and bpMRI E. % free PSA and bpMRI F. PSA and bpMRI**

**
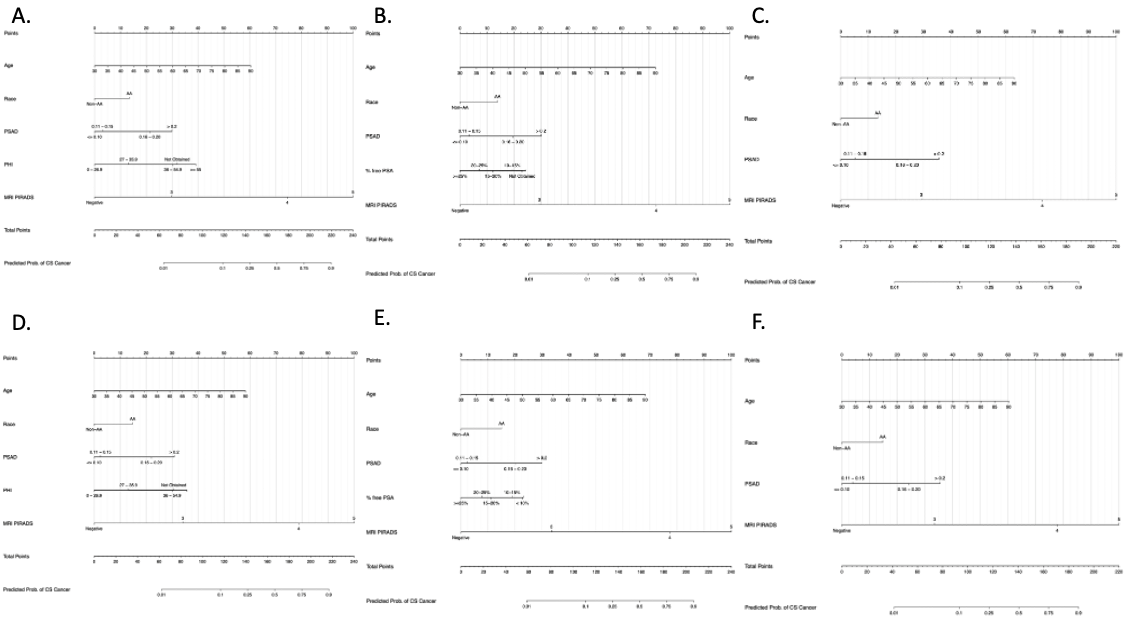
**

**Supplemental Figure 3: Receiver Operating Characteristic Curves for ≥GG2 PCa**





**Supplemental Figure 4: Receiver Operating Characteristic Curves for ≥GG3 PCa**


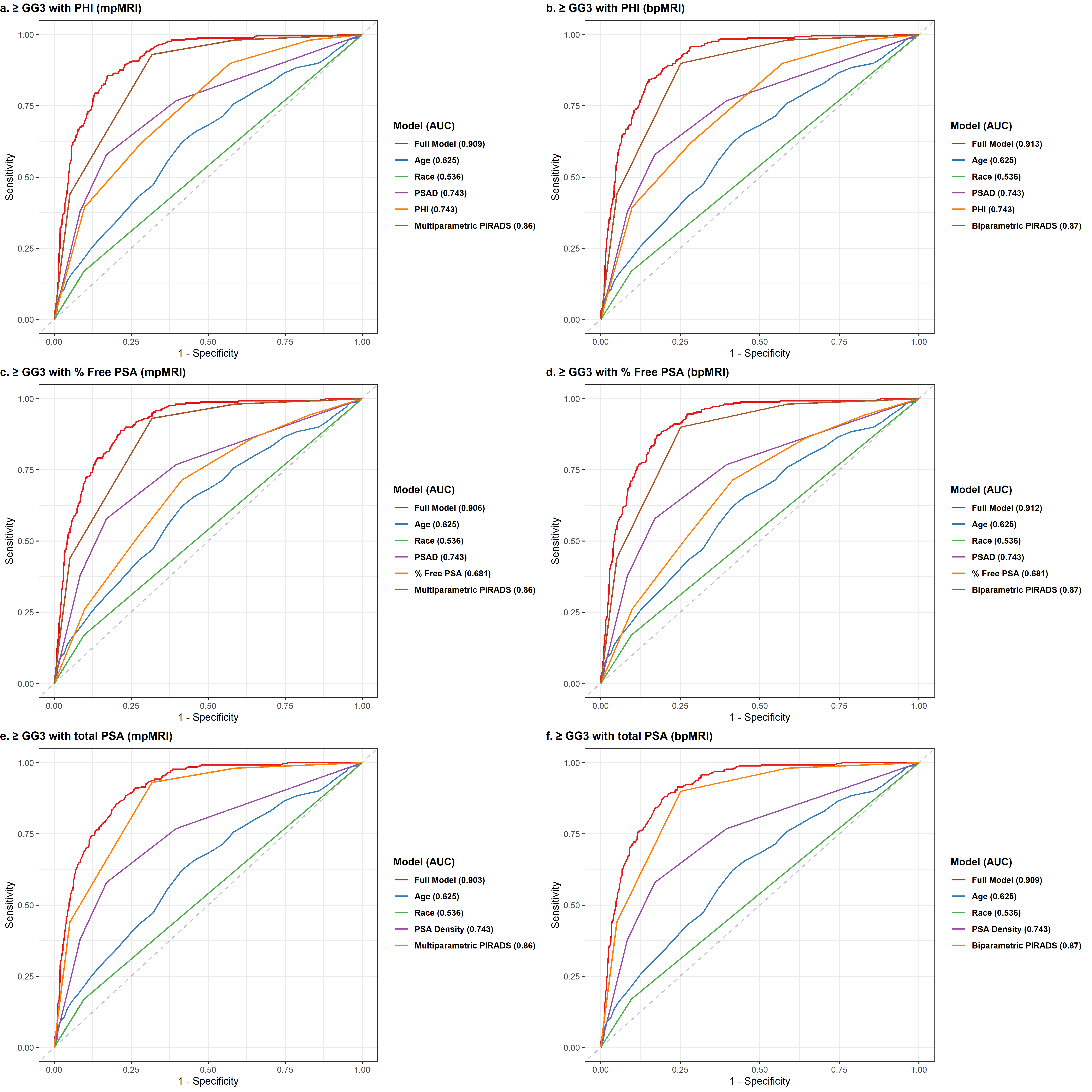


**Supplemental Figure 5: Decision Curve Analysis for ≥GG2 PCa**


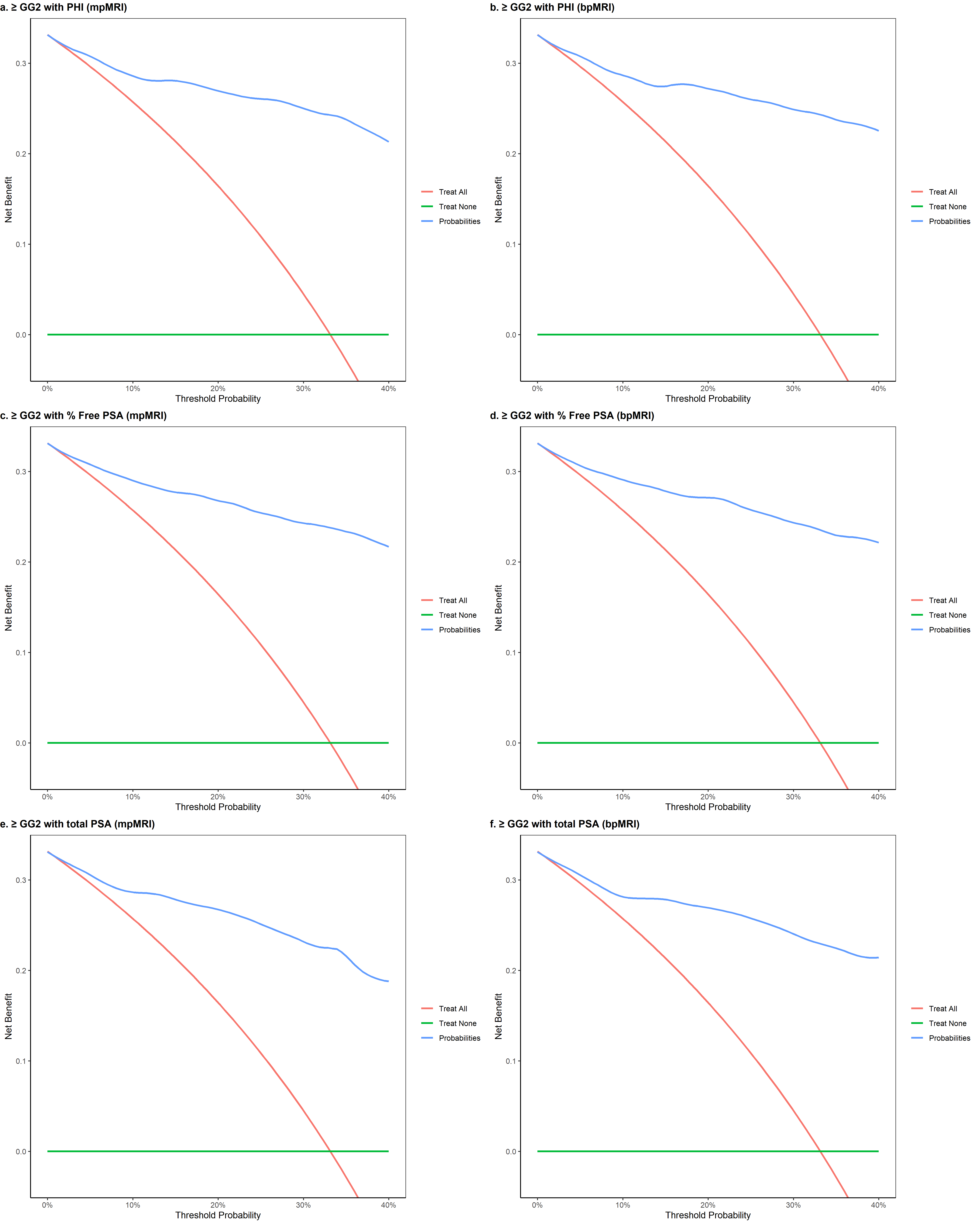


**Supplemental Figure 6: Decision Curve Analysis for ≥GG3 PCa**


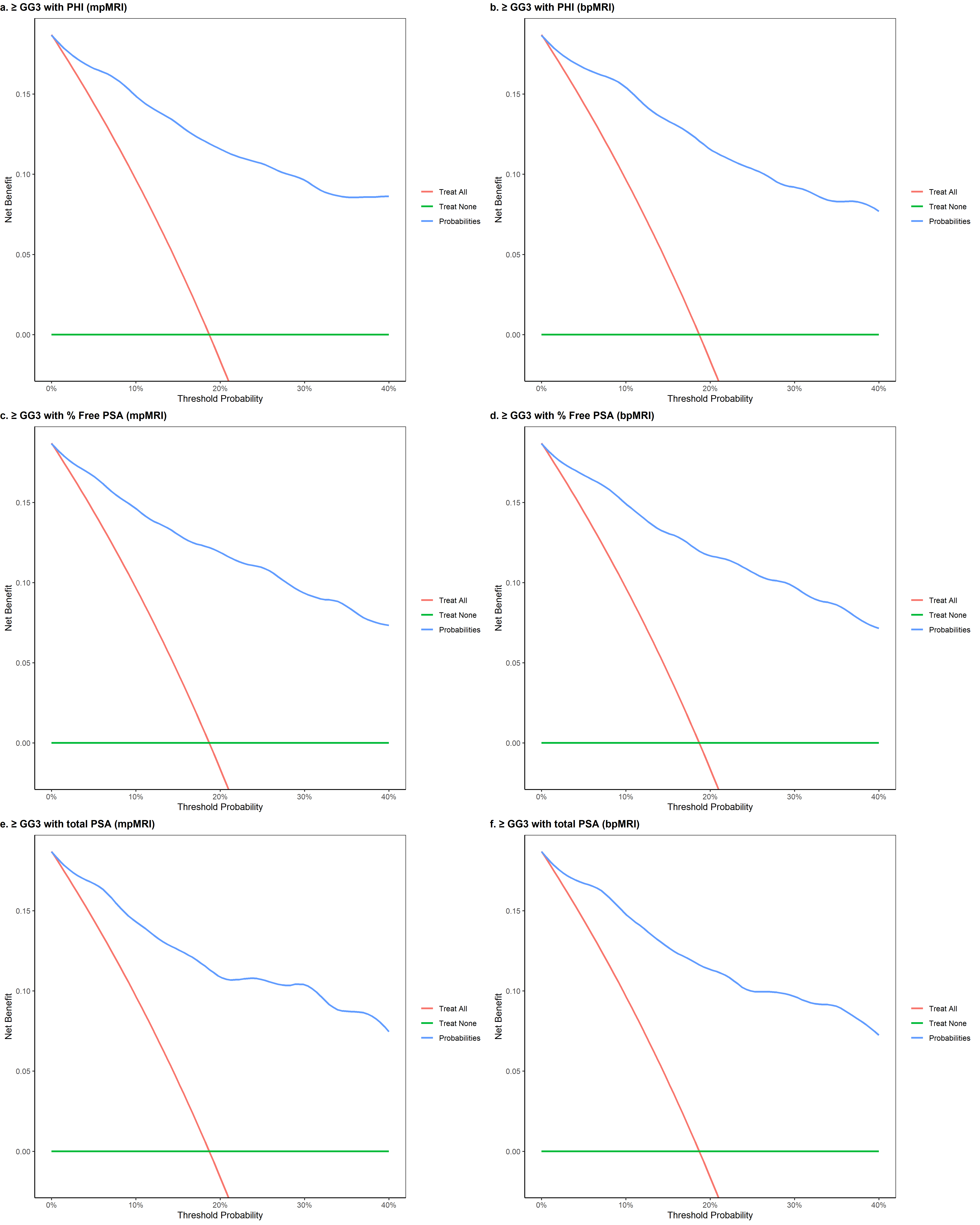


**Supplemental Figure 7: Calibration Curves for ≥GG2 PCa**
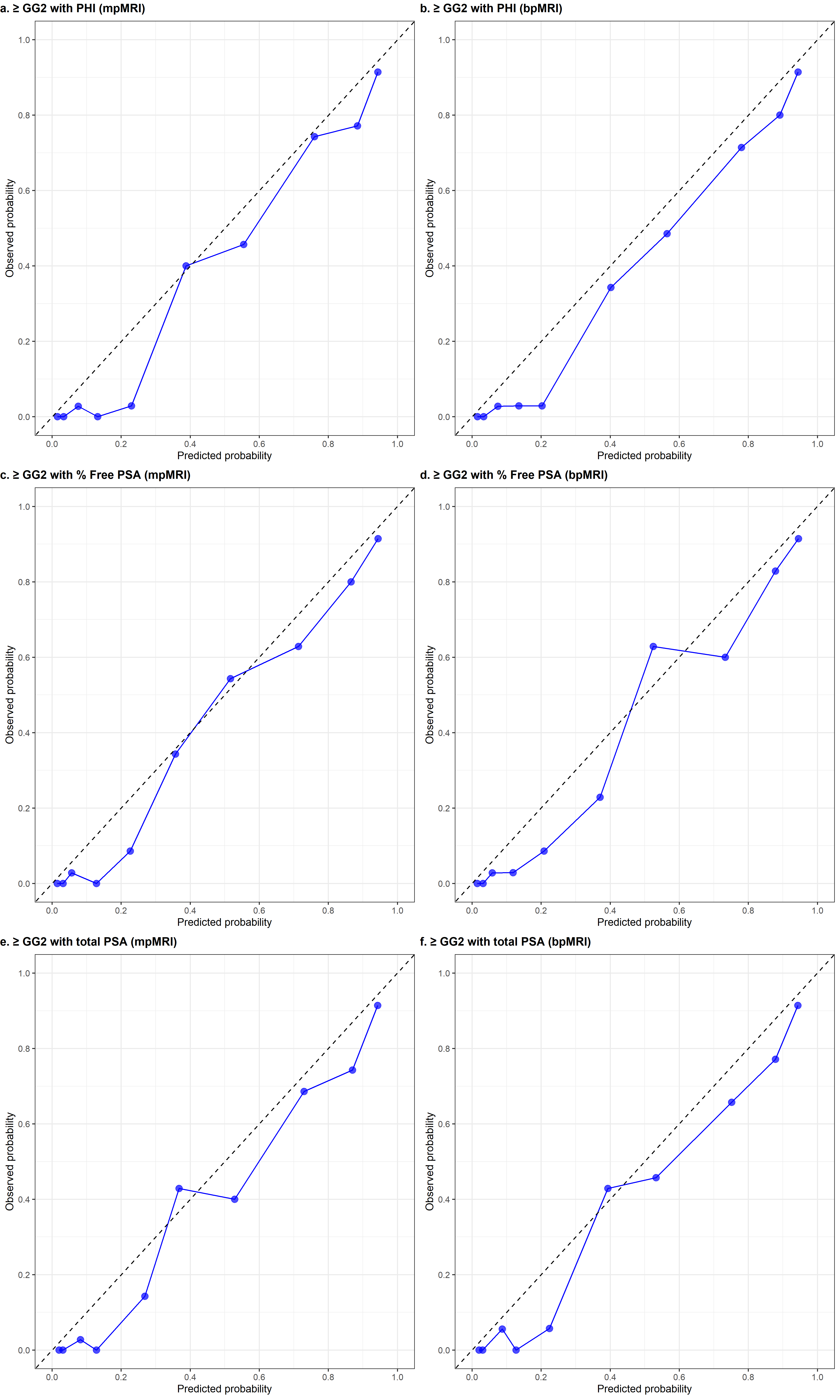


**Supplemental Figure 8: Calibration Curves for ≥GG3 PCa**


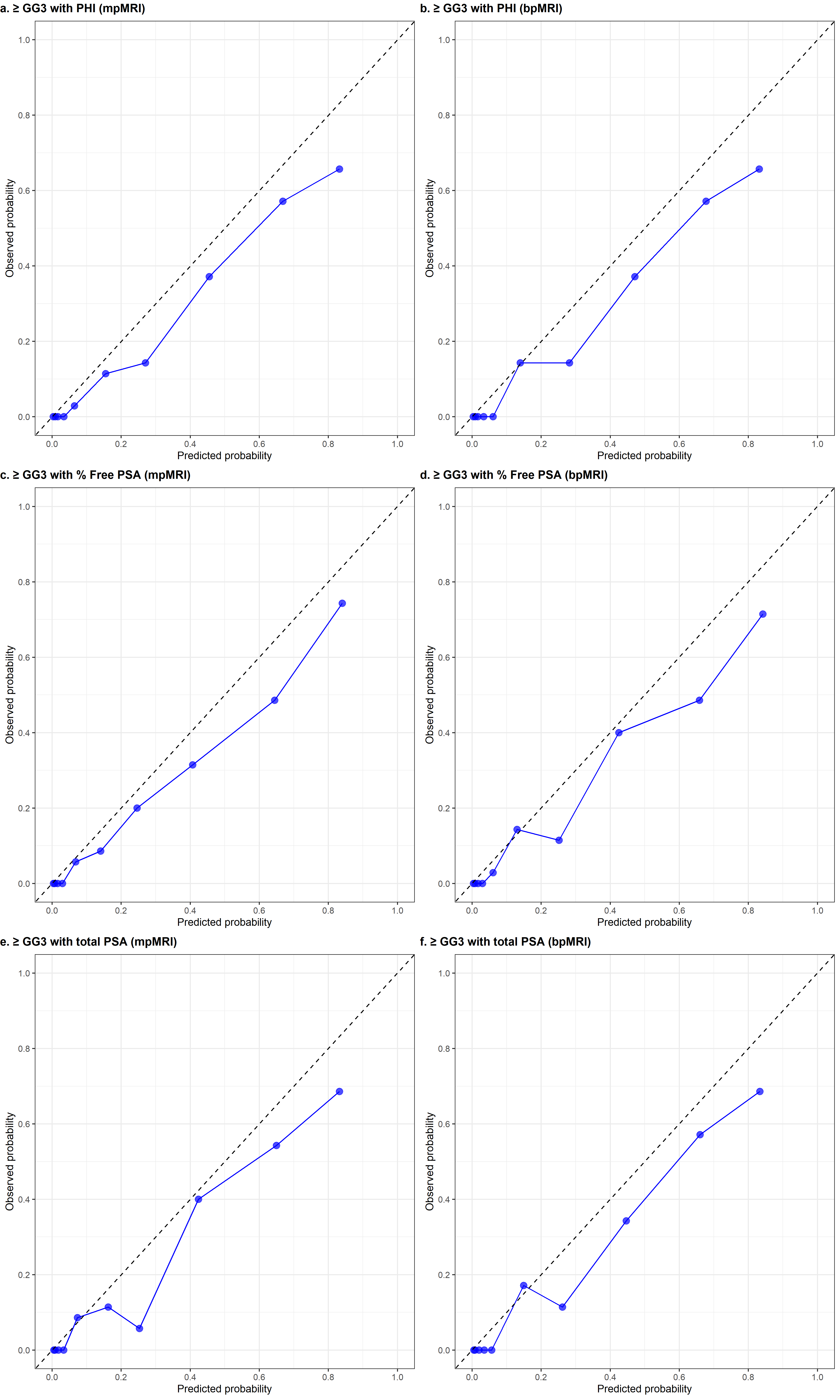

Supplement: Supplementary file 1 — Table S1: Multivariable Analysis of Factors Predictive of csPCa with PIRADS Classification by Biparametric MRI (PIRADS DWI 3, 4, and 5) as well as a fully Dynamic Contrast Enhancement stratified Model (3‐, 3+, 4‐, 4+, 5‐, 5+). Table S2: AUCs for original PIRADS, stratification by DCE, and bpMRI parameters. Table S3: Baseline Characteristics of Development Cohort for csPCa. Table S4: Baseline Characteristics of Development Cohort for ≥GG3 PCa. Table S5: Final Multivariable Model for ≥GG2 PCa with PHI for mpMRI. Table S6: Final Multivariable Model for ≥GG3 PCa with PHI for mpMRI. Table S7: Final Multivariable Model for ≥GG2 PCa with PHI for bpMRI. Table S8: Final Multivariable Model for ≥GG3 PCa with PHI for bpMRI. Table S9: Final Multivariable Model for ≥GG2 PCa with % free PSA for mpMRI. Table S10: Final Multivariable Model for ≥GG3 PCa with % free PSA for mpMRI. Table S11: Final Multivariable Model for ≥GG2 PCa with % free PSA for bpMRI. Table S12: Final Multivariable Model for ≥GG3 PCa with % free PSA for bpMRI. Table S13: Final Multivariable Model for ≥GG2 PCa with total PSA for mpMRI. Table S14: Final Multivariable Model for ≥GG3 PCa with total PSA for mpMRI. Table S15: Final Multivariable Model for ≥GG2 PCa with total PSA for bpMRI. Table S16: Final Multivariable Model for ≥GG3 PCa with total PSA for bpMRI. Table S17: DeLong comparison of ROC models for mpMRI and bpMRI models for development cohort. Table S18: Baseline Characteristics of Validation Cohort for ≥GG2 PCa. Table S19: Baseline Characteristics of Validation Cohort for ≥GG3 PCa. Table S20: Calibration Curve Test Characteristics. Figure S1: Nomograms for Advanced serum biomarker for ≥GG2 PCa A. PHI and mpMRI B. % free PSA and mpMRI C. PSA and mpMRI D. PHI and bpMRI E. % free PSA and bpMRI F. PSA and bpMRI. Figure S2: Nomograms for Advanced serum biomarker for ≥GG3 PCa A. PHI and mpMRI B. % free PSA and mpMRI C. PSA and mpMRI D. PHI and bpMRI E. % free PSA and bpMRI F. PSA and bpMRI. Figure S3: Receiver [file BCO2-5-865-s001.docx]
